# Supplementary material for: Zinc(II)—The Overlooked Éminence Grise of Chloroquine’s Fight against COVID-19?
Source: Pharmaceuticals (Basel). 2020 Sep 1;13(9):228. doi: 10.3390/ph13090228 (PMC7558363; doi:10.3390/ph13090228)
Supplement: Supplementary file 1 [file pharmaceuticals-13-00228-s001.pdf]

SUPPLEMENTARY INFORMATION

Zinc(II) - the overlooked éminence grise of chloroquine's fight against COVID-19?

Aleksandra Hecel [1,#], Małgorzata Ostrowska [1,#], Kamila Stokowa-Sołtys [1,#], Joanna Wątły [1,#], Dorota Dudek [1,#], Adriana Miller [1,#], Sławomir Potocki [1], Agnieszka Matera-Witkiewicz [2], Alicia Dominguez-Martin [3], Henryk Kozłowski [1,4] and Magdalena Rowińska-Żyrek [1,*]

[1] Faculty of Chemistry, University of Wroclaw, F. Joliot-Curie 14, 50-383 Wroclaw, Poland
[2] Screening Laboratory of Biological Activity Tests and Collection of Biological Material, Faculty of Pharmacy, Wroclaw Medical University, Borowska 211A, 50-556 Wroclaw, Poland
[3] Department of Inorganic Chemistry, Faculty of Pharmacy, University of Granada, E-18071 Granada, Spain
[4] Opole Medical School, Katowicka 68, 40-060 Opole, Poland

Table S1. Summary of non-CQ and non-HCQ based therapeutics in anti-COVID-19 clinical trials.

Drug	Therapeutic effect	Reference
Acetylcysteine	mucolytic drug used in acute and chronic bronchitis and other respiratory diseases with high mucosal secretion; moderates clinging mucous secretionsand enhanced glutathione S-transferase activity	[1]
Amlodipine	calcium channel blocker; indicated for the treatment of high blood pressure and chronic angina pectoris and angiospastic angina	[2,3]
Amoxicillin	β-lactam antibiotic; used to treat a number of bacterial infections such as acute sinusitis, acute cystisis and acute pyelonephritis	[4]
Anakinra	recombinant interleukin-1 receptorantagonist, immunosupresor; used in rheumatoid arthritistogether with methotrexate when self-methotrexate treatment response is insufficient; studies have shown that administration of anakinra reduces 28-day mortality by 30% in patients with severe sepsis and macrophage activation syndrome	[5]
Acetylsalicylic acid (Aspirin)	nonsteroidal anti-inflammatory drug with analgesic, antipyretic and anti-inflammatory effect, also platelets aggregation inhibitor; exhibits antiviral activityagainst influenza virus (NCT04343001); 9 CTs (in mono- or combination therapy with, e.g. clopidogrel, rivaxoban, ivermectin, losartan	[6,7]
Atorvastatin	HMG-CoA reductase inhibitor, hypolipemic drug; used for lowering cholesterol and in prevention of cardiovascular disease	[8]
Atovaquone	synthetic hydroxynaphthoquinone with antiprotozoal activity; used for the treatment of malariain combinationtherapy with proguanil hydrochloride	[9]
Aviptadil	synthetic form of vasoactive intestinal polypeptide (VIP); used for the treatment of ARDS; very promising in the treatment of COVID-19 patients with associated acute respiratory distress (NCT04311697)	[10]
Azithromycin	macrolide antibiotic; induces antivirial response in bronchial epithelial cells and may also be used to treat malaria;may be helpfulin treatingcoronaviral infections	[11,12]
Baricitinib	Janus kinases inhibitor; used for the treatment of rheumatoid arthritis as monotherapy or associated with methotrexate; in severe acute respiratory syndrome coronavirus 2 infection with ongoing pneumoniamaydecrease the hyperinflammation (prevention of lung damage)	[13–16]
BCG Vaccine	vaccine against tuberculosis; *in vitro* and *in vivo* studies have shown a beneficial effect also against various viral pathogens, e.g. respiratory syncytial, yellow fever, herpes simplex and human papilloma viruses	[17]
BLD-2660	inhibitor of calpain (CAPN) 1, 2, and 9; has a potential in reducing viral replication and nonproductive host-response to infection (the cause of morbidity and mortality in COVID-19), NCT04334460	[18]
Bromhexine	mucolytic drug; used for the treatment of respiratory disorders; has also a potential for the treatment of influenza virus and coronavirus infections. In a	[19]

	clinical trial (NCT04340349), it will be tested together with hydroxychloroquine (in low doses)	
Budesonide	glucocorticosteroid; used in bronchial asthma treatment, acute laryngitis, traecheitis, bronchi, also in chronic pulmonary disease to reduce inflammation; can suppress the immune reaction of the respiratory system locally	[20,21]
Calciferol	vitamin D; it is proposed that vitamin D, as a nutritional ergogenic aid, could be a potential intervention to fight against COVID-19 infected patients; bovine coronavirus studies have shown that reduced levels of vitamin D in calves have contributed to their infection (NCT04334005)	[22,23]
CamostatMesilate	protease inhibitor; used in remission of acute symptoms of chronic pancreatitis and postoperative reflux esophagitis; acts as an inhibitor of the TMPRSS2 host cell serine protease, which is needed to prepare viral S protein for entry into the cell (NCT04338906); reduces significantly the infection of Calu-3 lung cells by *SARS-CoV-2* (in vitro study)	[24,25]
Captopril	zinc ion-utilizing human angiotensin converting enzyme (ACE); mainly used to treat hypertension and kidney problems caused by diabetes	[26]
Ceftarolinefosamil	broad-spectrum oxyimino cephalosporin antibiotic with activity against respiratory pathogens including *S. pneumoniae*, *H. influenza*, *M. pneumoniae*, and influenza/parainfluenza viruses; used in non-hospital pneumonia	[27,28]
Ceftriaxone	widespectrum antimicrobial activity against both gram-positive and gram-negative organisms	[29]
Chlorhexidine	disinfectant and antiseptic agent; used e.g. as skin disinfectant; strong bactericidal and bacteriostatic effect;some *Pseudomonas*,*Proteus*, acid-resistant mycobacteria and spores are resistant to this agent	[30]
Chlorine dioxide	disinfectant; strong inhibitory effects on microbes and parasites	[31]
Ciclesonide	inhaled corticosteroid; used in chronic bronchial asthma; shows good antiviral activity against *SARS-CoV-2*; used also in mild *SARS-COV-2* infections in combination with hydroxychloroquine (NCT04330586)	[32]
Clevudine	antiviral drug; activity against hepatitis B virus (HBV)	[33]
Cobicistat	CYP3A inhibitor with no antiviral activity; indicated for *HIV*; used as a pharmacokinetic enhancer of certain antiviral agents (e.g. elvitegravir, atazanavir, and darunavir)	[34]
Colchicine	plant alkaloid; has some effect on the inflammasome NLP3 and microtubule formation which are associated with the pathogenetic cycle of coronavirus (NCT04326790)	[35,36]
Cyclosporine	immunosuppressant; used to help prevent rejection in organ transplant patients; it is also used to treat rheumatoid arthritis and psoriasis. In a clinical trial, it will be added to standard treatment in order to improve the prognosis of hospitalized patients with *SARS-COV-2* infections infections. The hypothesis of clinical study is that the cyclosporine is safe and it will have antiviral and anti-cytokine effects (NCT04412785)	[37,38]
Darunavir	protease inhibitor; used to treat HIV associated with ritonavir and cobicistat	[39]
DAS181	recombinant protein; antiviral agent; *in vitro* and *in vivo* studies have shown against parainfluenza and other viruses activity	[40–42]
Deferoxamine	chelator of ferric ion; used to treat transfusion related chronic iron overload; inhibits human *cytomegalovirus* replication	[43,44]
Defibrotide	oligonucleotide mixture with demonstrated antithrombotic, fibrinolytic, anti-adhesive and anti-inflammatory actions; used for hepatic veno-occlusive disease (VOD)	[45]
Dexamethasone	corticosteroid; anti-inflammatory, anti-allergic and immunosuppressive properties; also in chronic corticotherapy; it is being investigated for the treatment of COVID-19 due to the safe and effective activity of cortycosteroids (also dexamethason) in the prevention of acute respiratory distress syndrome (ARDS), including viral pneumonia (NCT04327401)	[46,47]
Diltiazem	calcium channel blocker; used to treat hypertension, angina pectoris and arterial	[48–50]

	hypertension; according to the data from Italy/USA, acute myocardial infraction with imposed respiratory failure significantly complicates the course of disease; used in the combination with thiazide, ACE inhibitors and ARB (NCT04330300)	
Doxycycline	strong and broad-spectrum inhibitor of matrix metalloproteases (MMPs); tetracycline-class antibiotic; used to treat bacterial infections, e.g. acne or urinary tract infections; may have also a role as an effective anti-viral agent and as an anti-inflammatory drug. Because it is know, that (i) the coronaviruses bind to metalloproteases (MMPs) of the host and (ii) the tetracyclines chelate the zinc from MMPs and their chelating activity can help inhibit COVID-19 infection by limiting of ability to the *SARS-CoV-2* virus replication, the primary aim of this study is to test whether doxycycline can benefit patients with severe acute respiratory syndrome coronavirus 2 infection (NCT04433078)	[51–53]
Eculizumab	monoclonal antibody, terminal complement inhibitor; used for treatment of atypical haemolyticuraemic syndrome and paroxysmal nocturnal haemoglobinuria	[54]
Enoxaparin	low molecular weight heparin; anticoagulant; used in angina pectoris andcardiac infraction; may play an anti-inflammatory role by reducing the release and biological activity of IL-6; investigated also as a preventing agent against *SARS-CoV-2* infection (NCT04345848)	[55–59]
Escin	mixture of saponins; has anti-inflammatory and anti-edematous effects; usually used to treat e.g. hemorrhoids, varicose veins, hematoma or venous congestion	[60]
Favipiravir	antiviral agent; selective and potent inhibitor of influenza viral RNA polymerase; used for the treatment of influenza; has a high potential activity against *SARS-CoV-2* virus; a few combination therapy CTsare actually performed with e.g. tocilizumab (NCT04310228), imatinib (NCT04356495), nitazoxanide and chloroquine (NCT04345419), hydroxychloroquine, azithromycin and zinc sulfate (NCT04373733)	[61,62]
Fluvoxamine	anti-depressant; used to treat obsessive-compulsive disorder; proposed to be used early in the course of the *SARS-CoV-2* infection to prevent more serious complications like shortness of breath (NCT04342663)	[63]
Folic Acid	type of vitamin B; used for production and maintenance new cells in the body and for prevention of the changes in DNA that can lead to cancer. It is suggested that folic acid may be used to inhibit the furin enzyme and may be useful in preventing or treating COVID-19 associated respiratory disease in the early stages of the disease	[64]
Galidesivir	antiviral drug; *in vitro* studies have shown activity against many viral pathogens and infectious agents including *MERS-CoV* and *SARS-CoV*	[65]
Giapreza	angiotensin II; used for increase blood pressure in adults with septic or other distributive shock	[66,67]
Hydrocortisone	corticosteroid; used for the treatment of*e.g.* allergic disorders, severe asthma skin conditions, ulcerative colitis, arthritis, lupus, multiple sclerosis, autoimmune etiology diseases,or lung disorders; in used in combination therapywith some antimicrobial agents, interferon beta and immunosupresors (NCT02735707)	[68,69]
Ibuprofen	non-steroidal anti- inflammatory drug; used for relief of rheumatic or muscular pain, back pain, and the pain of non-serious arthritic conditions, caused by swelling, stiffness and inflammation; evaluation of the reduction in severity and progression of lung injury in patients with *SARS-CoV-2* infections is under investigation/4th phase (NCT04334629)	[70,71]
Imatinib	tyrosine kinase inhibitor; has been approved for treatment of many hematologic and solid neoplasm; as a weak base may be cumulated in lysosymes and lead to some antiviral effects during alkalization of lysosymes required for virus/cell fusion; has activity against *SARS-CoV* (*in vitro*) and *MERS-CoV*, and demonstrates also anti-inflammatory activity; used in combination with lopinavir/ritonavir, baricitinib and hydroxychloroquine (NCT04346147)	[72–75]
Indomethacin	non-steroidal anti-inflammatory drug; used to reduce fever, pain, stiffness, and swelling from inflammation; has a potent antiviral activity against *SARS* coronavirus	[76,77]
Isotretinoin	retinoid (synthetic analog of vitamin A); used totreat severe cases of acne vulgaris; isotretinoin has been reported to increase CD4 counts and significantly	[78,79]

	reduces viremia in *HIV* patients with acne vulgaris; it is suggested that isotretoin therapy may affect the prevention of *SARS-COV-2* infection (NCT04361422)	
Ivermectin	anti-inflammatory drug,broad-spectrum anti-parasite medication; has also anti-viral activity against a broad range of viruses; inhibits the replication of *SARS-CoV-2 in vitro*; investigated also with hydroxychloroquine, nitazoxanide and favipiravir combination therapy	[80]
L-Ascorbic acid	vitamin C; mainly in intravenous use; may attenuate inflammation and vascular injury associated with sepsis and acute respiratory distress syndrome (ARDS); may also be effective in the treatment of COVID-19 pneumonia (NCT04323514)	[81–83]
Leronlimab (PRO 140)	monoclonal antibody, antiviral agent, belongs to a new class of HIV/AIDS therapeutics; used in CT of mild and critical *SARS-COV-2* infected patients	[84]
Levofloxacin	fluoroquinolone antibacterial agent; broad spectrum of activity against gram-positive andgram-negative bacteria also atypical respiratory pathogens (NCT02735707)	[85]
Linagliptin	DPP4 inhibitor; used to treat type 2 diabetes	[86,87]
Losartan	antihypertensive drug; angiotensin II receptor blocker; used to treat hypertension and renal disease; the main goal of one from many studies of losartanis identification whether or not ARBs have an impact on inhibiting the progression to respiratory failure in COVID-19 among patients with hipoxia (NCT04340557)	[26]
Mavrilimumab	human monoclonal antibody; investigated for the treatment of rheumatoid arthritis	[88,89]
Methylprednisolone	glucocorticosteroid; a drug with long-term anti-inflammatory and immunosuppressive effects; used in combination therapy with e.g. tacrolimus (NCT04341038) or siltuximab (NCT04329650)	[46,90]
Naproxen	non-steroidal, anti-inflammatory drug; inhibitor of both cyclooxygenase (COX-2) and of *influenza A* virus nucleoprotein (NP); study suggest that naproxen could combine a broad-spectrum antiviral activity with its well-known anti-inflammatory action that could help in reducing severe respiratory mortality associated with COVID-19	[91]
Nintedanib	inhibitor of tyrosine kinases; used in pulmonary fibrosis, systemic sclerosis-associated interstitial lung disease, and non-small cell lung cancer (NSCLC); actually efficacy and safety in the treatment of pulmonary fibrosis in patients with moderate to severe COVID-19(NCT04338802)	[92,93]
Nitazoxanide	thiazolide compound with antiparasitic, antibacterial and antiviral activity; inhibits a broad range of influenza A and B viruses, replication of a wide variety of other RNA and DNA viruses (e.g. respiratory syncytial virus, parainfluenza, coronavirus, rotavirus, norovirus, hepatitis B, hepatitis C, dengue, yellow fever) in cell culture assays; in CTs study in mono or combined therapy with e.g. ivermectin (NCT04351347, NCT04341493), hydroxychloroquine (NCT04341493) or azithromycin (NCT04382846)	[94–96]
Nitric Oxide inhaled nitric oxide gas (NO)	used for the relaxation of smooth muscle cells in the vasculature; has shown to have antiviral activity against other coronavirus strains (e.g. during the 2003 *SARS* outbreak); greatly increased the survival rate of infected eukaryotic cells, suggesting its direct antiviral effect.Currently, high doses of inhaled NO against *SARS-CoV-2* are studied (e.g. NCT04338828)	[97–99]
Nivolumab	monoclonal antibody; usually used to treat melanoma and many types of cancer, e.g. lung, kidney head and neck, urothelial, colon and liver. The purpose of clinical study (NCT04343144) is to show the efficacy of nivolumab to treat patients with COVID-19 (in combination with standard treatments)	[100]
Oseltamivir	antiviral drug; neuraminidase inhibitor; shows efficacy in the treatment of natural acquired influenza in humans, and might also protect people and animals from influenza; in over 15 CTs with different combination of drugs, e.g. hydrochloroquine and azitromycine (NCT04338698), lopinavir/ritonavir (NCT04255017), favipiravir (NCT04349241), antibody-rich plasma (NCT04348877) or arbidol (NCT04255017)	[101,102]
Peginterferon lambda 1a	type III interferon (IFN); stimulates immune response; high therapeutic potential in COVID-19 – λ interferon is effective against SARS coronavirus (genetic	[103,104]

	similarities between *SARS-CoV* and *SARS-CoV-2* are evident, NCT04344600)	
Pembrolizumab	monoclonal antibody; used to treat different types of cancer, e.g. lung or head and neck	[105–107]
Piclidenoson	adenosine A3 receptor inhibitor; anti-inflammatory agent; tested for plaque-type psoriasis	[108,109]
Piperacillin	beta-lactam antibiotic; in beta-lactamase inhibitor combination; broad spectrum of antibacterial activity also in pneumonia	[110]
Povidone-Iodine	antiseptic; used for hand disinfection, skin preparation and antiseptic irrigation; study hypothesis: oral gargles and nasal rinses using a povidone iodine solution will help to reduce the viral load in the nasopharynx and oropharynx	[111–114]
Prednisone	Corticosteroide ;anti-inflammatory and immunosuppressive agent; in COVID-19 treatment, the reducing influence on the risk of acute respiratory distress syndrome is tested (NCT04344288)	[115]
Remdesivir (GS-5734™)	nucleotide analog; broad-spectrum antiviral drug; currently available evidence about effects against coronaviruses are favorable (*in vivo* and *in vitro* study); 35 CTs – mono and combined therapy with e.g. hydroxychloroquine, lopinavir/ritonavir (NCT04330690), tocilizumab (NCT04409262) or baricitinib (NCT04401579)	[116,117]
Ribavirin	guanosine nucleoside; broad-spectrum virustatic activity; actually in 9 CTs, thre completed, one of them in combination therapy with lopinavir/ritonavir and IFN-β (NCT04276688)	[118–121]
Ritonavir	HIV protease inhibitor; in combination with other HIV therapies and antivirals, a potential treatment for COVID-19 caused by *SARS-CoV-2*, e.g. with xiyanping, (NCT04295551), danoprevir, (NCT04345276), ASC09 (NCT04261907) or interferon-β 1 and interferon-β 1b (NCT04343768); INF-β is even 10 times more effective than any other types of interferons tested against SARS and the most efficient antiviral drug against *SARS-CoV* so far), at the moment 80 CTs are performed in conjunction with e.g. danoprevir, lopinavir, hydroxychloroquine sulfate, interferon nebulization	[122–130]
Rivaroxaban	anticoagulant; direct factor Xa inhibitor; used for the prevention of venous thromboembolism in adults e.g. after knee replacement surgery or used to treatdeep vein thrombosis and pulmonary embolism;is studied in patients with COVID-19 as an anticoagulant agent (NCT04416048)	[131,132]
Ruxolitinib	JAK1 and JAK2 selective inhibitor using in primary marrow fibrosis; responsible for multiple cellular signals including the proinflammatory interleukin-6 (IL-6); study hypothesis: stop of the disregulated immune response caused by COVID-19 (NCT04334044)	[14]
Sargramostim	immunostimulator; stimulates white blood cell (WBC) production, and in particular, neutrophil, macrophage, and dendritic cell production; in CT the treatment in acute hypoxic respiratory failure due to COVID-19 is tested (NCT04326920)	[133]
Sarilumab	monoclonal antibody; inhibits the IL-6 receptor; and thereby may prevent of the overactive inflammatory response in the lungs of patients who are in severe or serious condition of COVID-19	[134]
Sildenafil citrate	PDE5 inhibitor; dilates blood vessels; COVID-19 study hypothesis: sildenafil citrate may help open the tiny vessels that draw oxygen from the lungs, allowing patients to overcome the respiratory distress that occurs in some cases of COVID-19 (NCT04304313)	[135]
Siltuximab	monoclonal antibody; anticancer drug, can be used as a treatment againstprostate or metastatic renal cells or cancer; the use of thisbiopharmaceutical at the time ofhyperinflammation episodes start, may improve the prognosis of the patients (NCT04329650)	[136]
Simvastatin	statin; used to lower the risk of cardiovascular disease and manage abnormal lipid levels; have anti-inflammatory and immunomodulatory effects	[137]
Sirolimus	mammalian target of rapamycin inhibitor; potent immunosuppressant and possesses both antifungal and antineoplastic properties	[138]
T89 (Dantonic)	botanical product, is a well-known traditional Chinese medicine; used for the treatment of e.g. angina pectoris (AP), myocardial infarction	[139]

Tacrolimus	immunosuppresor agent; used after organ transplant, in severe refractory uveitis, and the skin condition vitiligo	[140]
Telmisartan	angiotensin II type 1 receptor blocker; used to treat high blood pressure in monotherapy or in combination with other antihypertensive agents. Telmisartan has been proposed as an alternative treatment for COVID-19 patients prior to the development of ARDS (NCT04355936)	[141–143]
Thalidomide	anti-inflammatory, anti-fibrotic, anti-angiogenesis drug; effective in severe lung injury (e.g. against *H1N1* influenza); also used for the treatment of inflammation associated with Hansen's disease (leprosy) and as a chemotherapeutic agent for patients with multiple myeloma, purposes for which it was originally prescribed off-label	[144–146]
Tocilizumab	humanized monoclonal anti– interleukin 6 receptor antibody; it is hypothesized that it can reduce mortality in patients with severe COVID-19 prone to Cytokine-Release Syndrome (CRS) and ARDS Acute Respiratory Distress Syndrome (ARDS) (NCT04335071)	[147,148]
Tofacitinib	Janus kinase inhibitor; used to treat rheumatoid arthritis, psoriatic arthritis, ankylosing spondylitis, administration of tofacitinib in patients with symptomatic pneumonia pulmonary flogosisreductionis analyzed (NCT04332042)	[149–152]
Tradipitant	neurokinin 1 antagonist; has been used in trials studying the treatment and prevention of eczema, pruritus, gastroparesis, chronic pruritus, and atopic dermatitis	[153]
Tranexamic Acid (TXA)	blood clot stabilizer; used routinely for reduction of blood loss in surgery and trauma; inhibits conversion of plasminogen to plasmin and is proposed to be used against COVID-19 – plasmin, endogenous protease, acts on *SARS-CoV-2* virus by cleaving a newly inserted furin site in the S protein of the virus thus increasing infectivity and virulence (NCT04338074)	[154]
Umifenovir (Arbidol)	indole-based antiviral drug, dual-acting; used in prophylaxis and for the treatment of influenza and other respiratory infections; *in vitro* study have shown effect against coronaviruses	[155–157]
Valsartan	cardiovascular drug; AT1 angiotensin II receptor inhibitor; used to treat high blood pressure, cardiac infraction, heart failure, and diabetic kidney disease; may prevent the development of ARDS and avert morbidity and mortality (NCT04335786)	[158,159]

References

1. Mokhtari, V.; Afsharian, P.; Shahhoseini, M.; Kalantar, S.M.; Moini, A. A Review on Various Uses of N-Acetyl Cysteine. *Cell J.***2017**, *19*, 11–17, doi:10.22074/cellj.2016.4872.

2. Zisaki, A.; Miskovic, L.; Hatzimanikatis, V. Antihypertensive Drugs Metabolism: An Update to Pharmacokinetic Profiles and Computational Approaches. *Curr. Pharm. Des.***2014**, *21*, 806–822, doi:10.2174/1381612820666141024151119.

3. Fares, H.; DiNicolantonio, J.J.; O'Keefe, J.H.; Lavie, C.J. Amlodipine in hypertension: a first-line agent with efficacy for improving blood pressure and patient outcomes. *Open Heart***2016**, *3*, e000473, doi:10.1136/openhrt-2016-000473.

4. Akhavan, B.J.; Vijhani, P. Amoxicillin. https://www.ncbi.nlm.nih.gov/books/NBK482250/ (accessed on 15 April 2020).

5. Shakoory, B.; Carcillo, J.A.; Chatham, W.W.; Amdur, R.L.; Zhao, H.; Dinarello, C.A.; Cron, R.Q.; Opal, S.M. Interleukin-1 Receptor Blockade Is Associated With Reduced Mortality in Sepsis Patients With Features of Macrophage Activation Syndrome. *Crit. Care Med.***2016**, *44*, 275–281, doi:10.1097/CCM.0000000000001402.

6. Mazur, I.; Wurzer, W.J.; Ehrhardt, C.; Pleschka, S.; Puthavathana, P.; Silberzahn, T.; Wolff, T.; Planz, O.; Ludwig, S. Acetylsalicylic acid (ASA) blocks influenza virus propagation via its NF-?B-inhibiting activity. *Cell. Micobiol.***2007**, *9*, 1683–1694, doi:10.1111/j.1462-5822.2007.00902.x.

7. Glatthaar-Saalmüller, B.; Mair, K.H.; Saalmüller, A. Antiviral activity of aspirin against RNA viruses of the respiratory tract-an in vitro study. *Influenza Other Respir. Viruses***2017**, *11*, 85–92, doi:10.1111/irv.12421.

8. Ye, Y.-C.; Zhao, X.-L.; Zhang, S.-Y. Use of Atorvastatin in Lipid Disorders and Cardiovascular Disease in Chinese Patients. *Chin. Med. J.***2015**, *128*, 259–266, doi:10.4103/0366-6999.149226.

9. Nixon, G.L.; Moss, D.M.; Shone, A.E.; Lalloo, D.G.; Fisher, N.; O'Neill, P.M.; Ward, S.A.; Biagini, G.A. Antimalarial pharmacology and therapeutics of atovaquone. *J. Antimicrob. Chemother.***2013**, *68*, 977–985, doi:10.1093/jac/dks504.

10. Leuchte, H.H.; Baezner, C.; Baumgartner, R.A.; Bevec, D.; Bacher, G.; Neurohr, C.; Behr, J. Inhalation of vasoactive intestinal peptide in pulmonary hypertension. *Eur. Respir. J.***2008**, *32*, 1289–1294, doi:10.1183/09031936.00050008.

11. Menzel, M.; Akbarshahi, H.; Bjermer, L.; Uller, L. Azithromycin induces anti-viral effects in cultured bronchial epithelial cells from COPD patients. *Sci. Rep.***2016**, *6*, 1418, doi:10.1038/srep28698.

12. Gielen, V.; Johnston, S.L.; Edwards, M.R. Azithromycin induces anti-viral responses in bronchial epithelial cells. *Eur. Respir. J.***2010**, *36*, 646–654, doi:10.1183/09031936.00095809.

13. Richardson, P.; Griffin, I.; Tucker, C.; Smith, D.; Oechsle, O.; Phelan, A.; Stebbing, J. Baricitinib as potential treatment for 2019-nCoV acute respiratory disease. *Lancet***2020**, *395*, e30-e31, doi:10.1016/S0140-6736(20)30304-4.

14. Stebbing, J.; Phelan, A.; Griffin, I.; Tucker, C.; Oechsle, O.; Smith, D.; Richardson, P. COVID-19: combining antiviral and anti-inflammatory treatments. *Lancet Infect. Dis.***2020**, *20*, 400–402, doi:10.1016/S1473-3099(20)30132-8.

15. Bechman, K.; Subesinghe, S.; Norton, S.; Atzeni, F.; Galli, M.; Cope, A.P.; Winthrop, K.L.; Galloway, J.B. A systematic review and meta-analysis of infection risk with small molecule JAK inhibitors in rheumatoid arthritis. *Rheumatology (Oxford)***2019**, *58*, 1755–1766, doi:10.1093/rheumatology/kez087.

16. Favalli, E.G.; Biggioggero, M.; Maioli, G.; Caporali, R. Baricitinib for COVID-19: a suitable treatment? *Lancet Infect. Dis.***2020**, doi:10.1016/S1473-3099(20)30262-0.

17. Moorlag, S.J.C.F.M.; Arts, R.J.W.; van Crevel, R.; Netea, M.G. Non-specific effects of BCG vaccine on viral infections. *Clin. Microbiol. Infect.***2019**, *25*, 1473–1478, doi:10.1016/j.cmi.2019.04.020.

18. Blade Therapeutics. Blade Therapeutics Announces Initiation of Phase 1 Study of Lead Calpain Inhibitor BLD-2660 for Fibrosis. https://www.blademed.com/news/jul-17-2018/ (accessed on 15 April 2020).

19. Shen, L.W.; Mao, H.J.; Wu, Y.L.; Tanaka, Y.; Zhang, W. TMPRSS2: A potential target for treatment of influenza virus and coronavirus infections. *Biochimie***2017**, *142*, 1–10, doi:10.1016/j.biochi.2017.07.016.

20. Liao, M.; Liu, Y.; Yuan, J.; Wen, Y.; Xu, G.; Zhao, J.; Chen, L.; Li, J.; Wang, X.; Wang, F.; et al. The landscape of lung bronchoalveolar immune cells in COVID-19 revealed by single-cell RNA sequencing. 2020, medRxiv doi:10.1101/2020.02.23.20026690. https://www.medrxiv.org/content/10.1101/2020.02.23.20026690v1 (accessed on 15 April 2020).

21. Gruppen, M.P.; Bouts, A.H.; Jansen-van der Weide, M.C.; Merkus, M.P.; Zurowska, A.; Maternik, M.; Massella, L.; Emma, F.; Niaudet, P.; Cornelissen, E.A.M.; et al. A randomized clinical trial indicates that levamisole increases the time to relapse in children with steroid-sensitive idiopathic nephrotic syndrome. *Kidney Int.***2018**, *93*, 510–518, doi:10.1016/j.kint.2017.08.011.

22. Greiller, C.; Martineau, A. Modulation of the Immune Response to Respiratory Viruses by Vitamin D. *Nutrients***2015**, *7*, 4240–4270, doi:10.3390/nu7064240.

23. Oma, V.S.; Tråvén, M.; Alenius, S.; Myrmel, M.; Stokstad, M. Bovine coronavirus in naturally and experimentally exposed calves; viral shedding and the potential for transmission. *Virol. J.***2016**, *13*, 123, doi:10.1186/s12985-016-0555-x.

24. Kawase, M.; Shirato, K.; van der Hoek, L.; Taguchi, F.; Matsuyama, S. Simultaneous Treatment of Human Bronchial Epithelial Cells with Serine and Cysteine Protease Inhibitors Prevents Severe Acute Respiratory Syndrome Coronavirus Entry. *J. Virol.***2012**, *86*, 6537–6545, doi:10.1128/JVI.00094-12.

25. Hoffmann, M.; Kleine-Weber, H.; Schroeder, S.; Krüger, N.; Herrler, T.; Erichsen, S.; Schiergens, T.S.; Herrler, G.; Wu, N.-H.; Nitsche, A.; et al. SARS-CoV-2 Cell Entry Depends on ACE2 and TMPRSS2 and Is Blocked by a Clinically Proven Protease Inhibitor. *Cell***2020**, *181*, 271-280.e8, doi:10.1016/j.cell.2020.02.052.

26. Ripley, E.; Hirsch, A. Fifteen years of losartan: what have we learned about losartan that can benefit chronic kidney disease patients? *Int. J. Nephrol. Renovasc. Dis.* **2010**, *3*, 93–98, doi:10.2147/ijnrd.s7038.

27. Frampton, J.E. Ceftaroline Fosamil: A Review of its Use in the Treatment of Complicated Skin and Soft Tissue Infections and Community-Acquired Pneumonia. *Drugs* **2013**, *73*, 1067–1094, doi:10.1007/s40265-013-0075-6.

28. Steed, M.E.; Rybak, M.J. Ceftaroline: A New Cephalosporin with Activity Against Resistant Gram-Positive Pathogens. *Pharmacotherapy* **2010**, *30*, 375–389, doi:10.1592/phco.30.4.375.

29. Li, X.; Li, H.; Li, S.; Zhu, F.; Kim, D.J.; Xie, H.; Li, Y.; Nadas, J.; Oi, N.; Zykova, T.A.; et al. Ceftriaxone, an FDA-approved cephalosporin antibiotic, suppresses lung cancer growth by targeting Aurora B. *Carcinogenesis* **2012**, *33*, 2548–2557, doi:10.1093/carcin/bgs283.

30. McDonnell, G.; Russell, A.D. Antiseptics and Disinfectants: Activity, Action, and Resistance. *Clin. Microbiol. Rev.* **1999**, *12*, 147–179, doi:10.1128/CMR.12.1.147.

31. Zhu, Z.; Guo, Y.; Yu, P.; Wang, X.; Zhang, X.; Dong, W.; Liu, X.; Guo, C. Chlorine dioxide inhibits the replication of porcine reproductive and respiratory syndrome virus by blocking viral attachment. *Infect. Genet. Evol.* **2019**, *67*, 78–87, doi:10.1016/j.meegid.2018.11.002.

32. Matsuyama, S.; Kawase, M.; Nao, N.; Shirato, K.; Ujike, M.; Kamitani, W.; Shimojima, M.; Fukushi, S. The inhaled corticosteroid ciclesonide blocks coronavirus RNA replication by targeting viral NSP15. 2020, medRxiv doi: 10.1101/2020.03.11.987016. https://www.biorxiv.org/content/10.1101/2020.03.11.987016v1 (accessed on 15 April 2020).

33. Yoo, B.C.; Kim, J.H.; Chung, Y.-H.; Lee, K.S.; Paik, S.W.; Ryu, S.H.; Han, B.H.; Han, J.-Y.; Byun, K.S.; Cho, M.; et al. Twenty-four-week clevudine therapy showed potent and sustained antiviral activity in HBeAg-positive chronic hepatitis B. *Hepatology* **2007**, *45*, 1172–1178, doi:10.1002/hep.21629.

34. Deeks, E.D. Cobicistat: A Review of Its Use as a Pharmacokinetic Enhancer of Atazanavir and Darunavir in Patients with HIV-1 Infection. *Drugs* **2014**, *74*, 195–206, doi:10.1007/s40265-013-0160-x.

35. Kurek, J. Cytotoxic Colchicine Alkaloids: From Plants to Drugs. In *Cytotoxicity*; Çelik, T.A., Ed.; InTech, 2018, ISBN 978-1-78923-430-5.

36. Deftereos, S.G.; Siasos, G.; Giannopoulos, G.; Vrachatis, D.A.; Angelidis, C.; Giotaki, S.G.; Gargalianos, P.; Giamarellou, H.; Gogos, C.; Daikos, G.; et al. The Greek study in the effects of colchicine in COvid-19 complications prevention (GRECCO-19 study): Rationale and study design. *Hellenic J. Cardiol.* **2020**, doi:10.1016/j.hjc.2020.03.002.

37. Tedesco, D.; Haragsim, L. Cyclosporine: a review. *J. Transplant.* **2012**, *2012*, 230386, doi:10.1155/2012/230386.

38. Lothar Faerber; Matthias Braeutigam; Gottfried Weidinger; Ulrich Mrowietz; Enno Christophers; H. J. Schulze; G. Mahrle; Hans Meffert; Sabine Drechsler. Cyclosporine in Severe Psoriasis. *Am J Clin Dermatol* **2001**, *2*, 41–47, doi:10.2165/00128071-200102010-00007.

39. Lascar, R.; Benn, P. Role of darunavir in the management of HIV infection. *HIV AIDS (Auckl)* **2009**, 31–39, doi:10.2147/hiv.s5397.

40. Salvatore, M.; Satlin, M.J.; Jacobs, S.E.; Jenkins, S.G.; Schuetz, A.N.; Moss, R.B.; van Besien, K.; Shore, T.; Soave, R. DAS181 for Treatment of Parainfluenza Virus Infections in Hematopoietic Stem Cell Transplant Recipients at a Single Center. *Biol. Blood Marrow Transplant.* **2016**, *22*, 965–970, doi:10.1016/j.bbmt.2016.02.011.

41. Jones, B.G.; Hayden, R.T.; Hurwitz, J.L. Inhibition of primary clinical isolates of human parainfluenza virus by DAS181 in cell culture and in a cotton rat model. *Antiviral Res.* **2013**, *100*, 562–566, doi:10.1016/j.antiviral.2013.09.014.

42. Malakhov, M.P.; Aschenbrenner, L.M.; Smee, D.F.; Wandersee, M.K.; Sidwell, R.W.; Gubareva, L.V.; Mishin, V.P.; Hayden, F.G.; Kim, D.H.; Ing, A.; et al. Sialidase Fusion Protein as a Novel Broad-Spectrum Inhibitor of Influenza Virus Infection. *Antimicrob. Agents Chemother.* **2006**, *50*, 1470–1479, doi:10.1128/AAC.50.4.1470-1479.2006.

43. Georgiou, N.A.; van der Bruggen, T.; Oudshoorn, M.; Nottet, H.S.L.M.; Marx, J.J.M.; van Asbeck, B.S. Inhibition of Human Immunodeficiency Virus Type 1 Replication in Human Mononuclear Blood Cells by the Iron Chelators Deferoxamine, Deferiprone, and Bleomycin. *J. Infect. Dis.* **2000**, *181*, 484–490, doi:10.1086/315223.

44. Wang, H.; Li, Z.; Niu, J.; Xu, Y.; Ma, L.; Lu, A.; Wang, X.; Qian, Z.; Huang, Z.; Jin, X.; et al. Antiviral effects of ferric ammonium citrate. *Cell Discov.* **2018**, *4*, 344, doi:10.1038/s41421-018-0013-6.

45. Pescador, R.; Capuzzi, L.; Mantovani, M.; Fulgenzi, A.; Ferrero, M.E. Defibrotide: Properties and clinical use of an old/new drug. *Vascul. Pharmacol.***2013**, *59*, 1–10, doi:10.1016/j.vph.2013.05.001.

46. Coutinho, A.E.; Chapman, K.E. The anti-inflammatory and immunosuppressive effects of glucocorticoids, recent developments and mechanistic insights. *Mol. Cell. Endocrinol.***2011**, *335*, 2–13, doi:10.1016/j.mce.2010.04.005.

47. Khilnani, G.C.; Hadda, V. Corticosteroids and ARDS: A review of treatment and prevention evidence. *Lung India***2011**, *28*, 114, doi:10.4103/0970-2113.80324.

48. Clerkin, K.J.; Fried, J.A.; Raikhelkar, J.; Sayer, G.; Griffin, J.M.; Masoumi, A.; Jain, S.S.; Burkhoff, D.; Kumaraiah, D.; Rabbani, L.; et al. Coronavirus Disease 2019 (COVID-19) and Cardiovascular Disease. *Circulation***2020**, *58*, 686, doi:10.1161/CIRCULATIONAHA.120.046941.

49. Zheng, Y.-Y.; Ma, Y.-T.; Zhang, J.-Y.; Xie, X. COVID-19 and the cardiovascular system. *Nat. Rev. Cardiol.***2020**, *17*, 259–260, doi:10.1038/s41569-020-0360-5.

50. Inciardi, R.M.; Lupi, L.; Zaccone, G.; Italia, L.; Raffo, M.; Tomasoni, D.; Cani, D.S.; Cerini, M.; Farina, D.; Gavazzi, E.; et al. Cardiac Involvement in a Patient With Coronavirus Disease 2019 (COVID-19). *JAMA Cardiol.***2020**, doi:10.1001/jamacardio.2020.1096.

51. Geisler, W.M.; Uniyal, A.; Lee, J.Y.; Lensing, S.Y.; Johnson, S.; Perry, R.C.W.; Kadrnka, C.M.; Kerndt, P.R. Azithromycin versus Doxycycline for Urogenital Chlamydia trachomatis Infection. *N. Engl. J. Med.***2015**, *373*, 2512–2521, doi:10.1056/NEJMoa1502599.

52. Yates, P.A.; Leone, A.M.; Reichel, E. A Proposed Randomized, Double Blind, Placebo Controlled Study Evaluating Doxycycline for the Prevention of COVID-19 Infection and Disease In Healthcare Workers with Ongoing High Risk Exposure to COVID-19. https://doi.org/10.1101/2020.05.11.20098525 (accessed on 7 July 2020).

53. Malek, A.E.; Granwehr, B.P.; Kontoyiannis, D.P. Doxycycline as a potential partner of COVID-19 therapies. *IDCases***2020**, *21*, e00864, doi:10.1016/j.idcr.2020.e00864.

54. Zelek, W.M.; Taylor, P.R.; Morgan, B.P. Development and characterisation of novel anti-C5 monoclonal antibodies capable of inhibiting complement in multiple species. *Immunology***2019**, *157*, doi:10.1111/imm.13083.

55. Shi, C.; Wang, C.; Wang, H.; Yang, C.; Cai, F.E.I.; Zeng, F.; Cheng, F.; Liu, Y.; Zhou, T.; Deng, B.I.N.; et al. The potential of low molecular weight heparin to mitigate cytokine storm in severe COVID-19 patients: a retrospective clinical study. 2020, medRxiv doi: 10.1101/2020.03.28.20046144. https://www.medrxiv.org/content/10.1101/2020.03.28.20046144v3.

56. Camprubí-Rimblas, M.; Tantinyà, N.; Bringué, J.; Guillamat-Prats, R.; Artigas, A. Anticoagulant therapy in acute respiratory distress syndrome. *Ann. Transl. Med.***2018**, *6*, 36, doi:10.21037/atm.2018.01.08.

57. Thachil, J. The versatile heparin in COVID-19. *J. Thromb. Haemost.***2020**, doi:10.1111/jth.14821.

58. Qian, Y.; Xie, H.; Tian, R.; Yu, K.; Wang, R. Efficacy of Low Molecular Weight Heparin in Patients with Acute Exacerbation of Chronic Obstructive Pulmonary Disease Receiving Ventilatory Support. *COPD***2014**, *11*, 171–176, doi:10.3109/15412555.2013.831062.

59. Li, X.; Ma, Y.; Chen, T.; Tang, J.; Ma, X. Unfractionated heparin inhibits lipopolysaccharide-induced expression of chemokines in human endothelial cells through nuclear factor-KB signaling pathway. *Zhonghua Wei Zhong Bing Ji Jiu Yi Xue***2016**, *28*, 117–121, doi:10.3760/cma.j.issn.2095-4352.2016.02.007.

60. Gallelli, L. Escin: a review of its anti-edematous, anti-inflammatory, and venotonic properties. *Drug Des. Devel. Ther.***2019**, *13*, 3425–3437, doi:10.2147/DDDT.S207720.

61. Furuta, Y.; Komeno, T.; Nakamura, T. Favipiravir (T-705), a broad spectrum inhibitor of viral RNA polymerase. *Proc. Jpn. Acad., Ser. B, Phys. Biol. Sci.***2017**, *93*, 449–463, doi:10.2183/pjab.93.027.

62. Cai, Q.; Yang, M.; Liu, D.; Chen, J.; Shu, D.; Xia, J.; Liao, X.; Gu, Y.; Cai, Q.; Yang, Y.; et al. Experimental Treatment with Favipiravir for COVID-19: An Open-Label Control Study. *Engineering***2020**, doi:10.1016/j.eng.2020.03.007.

63. Rosen, D.A.; Seki, S.M.; Fernández-Castañeda, A.; Beiter, R.M.; Eccles, J.D.; Woodfolk, J.A.; Gaultier, A. Modulation of the sigma-1 receptor–IRE1 pathway is beneficial in preclinical models of inflammation and sepsis. *Sci. Transl. Med.***2019**, *11*, eaau5266, doi:10.1126/scitranslmed.aau5266.

64. Sheybani, Z.; Dokoohaki, M.H.; Negahdaripour, M.; Dehdashti, M.; Zolghadr, H.; Moghadami, M.; Masoompour, S.M.; Zolghadr, A.R. The Role of Folic Acid in the Management of Respiratory Disease Caused by COVID-19.

https://chemrxiv.org/articles/The_Role_of_Folic_Acid_in_the_Management_of_Respiratory_Disease_Caused_by_COVID-19/12034980/1 (accessed on 7 July 2020).

65. Taylor, R.; Kotian, P.; Warren, T.; Panchal, R.; Bavari, S.; Julander, J.; Dobo, S.; Rose, A.; El-Kattan, Y.; Taubenheim, B.; et al. BCX4430 – A broad-spectrum antiviral adenosine nucleoside analog under development for the treatment of Ebola virus disease. *J. Infect. Public Health* **2016**, *9*, 220–226, doi:10.1016/j.jiph.2016.04.002.

66. Wang, H.; Das, S.; Wieruszewski, P.M.; Taji, J.; Bartlett, B.; Azad, N.; Chowdhury, A.; Kolar, G.J.; Jain, N.; Subla, M.R.; et al. Unexpected BP Sensitivity to Angiotensin II in a Patient With Coronavirus Disease 2019, ARDS, and Septic Shock. *Chest* **2020**, doi:10.1016/j.chest.2020.04.015.

67. Zangrillo, A.; Landoni, G.; Beretta, L.; Morselli, F.; Serpa Neto, A.; Bellomo, R. Angiotensin II infusion in COVID-19-associated vasodilatory shock: a case series. *Crit. Care* **2020**, *24*, 227, doi:10.1186/s13054-020-02928-0.

68. Jantz, M.A.; Sahn, S.A. Corticosteroids in acute respiratory failure. *Am. J. Respir. Crit. Care Med.* **1999**, *160*, 1079–1100, doi:10.1164/ajrccm.160.4.9901075.

69. Ferrara, G.; Petrillo, M.; Giani, T.; Marrani, E.; Filippeschi, C.; Oranges, T.; Simonini, G.; Cimaz, R. Clinical Use and Molecular Action of Corticosteroids in the Pediatric Age. *Int. J. Mol. Sci.* **2019**, *20*, 444, doi:10.3390/ijms20020444.

70. Bierma-Zeinstra, S.M.A.; Brew, J.; Stoner, K.; Wilson, R.; Kilbourn, A.; Conaghan, P.G. A new lipid formulation of low dose ibuprofen shows non-inferiority to high dose standard ibuprofen: the FLARE study (flaring arthralgia relief evaluation in episodic flaring knee pain) – a randomised double-blind study. *Osteoarthr. Cartil.* **2017**, *25*, 1942–1951, doi:10.1016/j.joca.2017.09.002.

71. Kennedy, T.P.; Rao, N.V.; Noah, W.; Michael, J.R.; Jafri, M.H.; Gurtner, G.H.; Hoidal, J.R. Ibuprofen prevents oxidant lung injury and in vitro lipid peroxidation by chelating iron. *J. Clin. Invest.* **1990**, *86*, 1565–1573, doi:10.1172/JCI114876.

72. Rizzo, A.N.; Sammani, S.; Esquinca, A.E.; Jacobson, J.R.; Garcia, J.G.N.; Letsiou, E.; Dudek, S.M. Imatinib attenuates inflammation and vascular leak in a clinically relevant two-hit model of acute lung injury. *Am. J. Physiol. Lung Cell. Mol. Physiol.* **2015**, *309*, L1294-304, doi:10.1152/ajplung.00031.2015.

73. Horne, G.A.; Stobo, J.; Kelly, C.; Mukhopadhyay, A.; Latif, A.L.; Dixon-Hughes, J.; McMahon, L.; Cony-Makhoul, P.; Byrne, J.; Smith, G.; et al. A randomised phase II trial of hydroxychloroquine and imatinib versus imatinib alone for patients with chronic myeloid leukaemia in major cytogenetic response with residual disease. *Leukemia* **2020**, *34*, 1775–1786, doi:10.1038/s41375-019-0700-9.

74. Wolf, A.M.; Wolf, D.; Rumpold, H.; Ludwiczek, S.; Enrich, B.; Gastl, G.; Weiss, G.; Tilg, H. The kinase inhibitor imatinib mesylate inhibits TNF-{alpha} production in vitro and prevents TNF-dependent acute hepatic inflammation. *Proc. Natl. Acad. Sci. U. S. A.* **2005**, *102*, 13622–13627, doi:10.1073/pnas.0501758102.

75. Wang, D.-Y.; Guo, J.-M.; Yang, Z.-Z.; You, Y.; Chen, Z.; Chen, S.-M.; Cheng, H.; Zhang, Y.-S.; Jiang, D.-Z.; Zuo, X.-L.; et al. The First Report of the Prevalence of COVID-19 in Chronic Myelogenous Leukemia Patients in the Core Epidemic Area of China: A Multicentre, Cross-Sectional Survey. https://www.medrxiv.org/content/10.1101/2020.03.12.20034876v2 (accessed on 7 July 2020).

76. Russell, B.; Moss, C.; Rigg, A.; van Hemelrijck, M. COVID-19 and treatment with NSAIDs and corticosteroids: should we be limiting their use in the clinical setting? *Ecancermedicalscience* **2020**, *14*, 1023, doi:10.3332/ecancer.2020.1023.

77. Amici, C.; Di Caro, A.; Ciucci, A.; Chiappa, L.; Castilletti, C.; Martella, V.; Decaro, N.; Buonavoglia, C.; Capobianchi, M.R.; Santoro, M.G. Indomethacin has a potent antiviral activity against SARS coronavirus. *Antivir. Ther. (Lond.)* **2006**, *11*, 1021–1030.

78. Abdelmaksoud, A.; Vestita, M.; El-Amawy, H.S.; Ayhan, E.; An, İ.; Öztürk, M.; Goldust, M. Systemic isotretinoin therapy in the era of COVID-19. *Dermatol. Ther.* **2020**, e13482, doi:10.1111/dth.13482.

79. Hamouda Elgarhy, L. Could patients taking isotretinoin therapy be immune against SARS-CoV-2? *Dermatol. Ther.* **2020**, e13573, doi:10.1111/dth.13573.

80. Caly, L.; Druce, J.D.; Catton, M.G.; Jans, D.A.; Wagstaff, K.M. The FDA-approved drug ivermectin inhibits the replication of SARS-CoV-2 in vitro. *Antiviral Res.* **2020**, *178*, 104787, doi:10.1016/j.antiviral.2020.104787.

81. Kashiouris, M.G.; L'Heureux, M.; Cable, C.A.; Fisher, B.J.; Leichtle, S.W.; Fowler, A.A. The Emerging Role of Vitamin C as a Treatment for Sepsis. *Nutrients* **2020**, *12*, doi:10.3390/nu12020292.

82. Fowler, A.A.; Truwit, J.D.; Hite, R.D.; Morris, P.E.; DeWilde, C.; Priday, A.; Fisher, B.; Thacker, L.R.; Natarajan, R.; Brophy, D.F.; et al. Effect of Vitamin C Infusion on Organ Failure and Biomarkers of Inflammation and Vascular Injury in Patients With Sepsis and Severe Acute Respiratory Failure: The CITRIS-ALI Randomized Clinical Trial. *JAMA*2019, *322*, 1261–1270, doi:10.1001/jama.2019.11825.

83. Carr, A.C.; Maggini, S. Vitamin C and Immune Function. *Nutrients***2017**, *9*, doi:10.3390/nu9111211.

84. Kaplon, H.; Reichert, J.M. Antibodies to watch in 2019. *MAbs***2019**, *11*, 219–238, doi:10.1080/19420862.2018.1556465.

85. Anderson, V.R.; Perry, C.M. Levofloxacin : a review of its use as a high-dose, short-course treatment for bacterial infection. *Drugs***2008**, *68*, 535–565, doi:10.2165/00003495-200868040-00011.

86. Iacobellis, G. COVID-19 and diabetes: Can DPP4 inhibition play a role? *Diabetes Res. Clin. Pract.***2020**, *162*, 108125, doi:10.1016/j.diabres.2020.108125.

87. Yamadera, S.; Nakamura, Y.; Inagaki, M.; Kenmotsu, S.; Nohara, T.; Sato, N.; Oguchi, T.; Tsuji, M.; Ohsawa, I.; Gotoh, H.; et al. Linagliptin inhibits lipopolysaccharide-induced inflammation in human U937 monocytes. *Inflamm. Regen.***2018**, *38*, 13, doi:10.1186/s41232-018-0071-z.

88. Burmester, G.R.; McInnes, I.B.; Kremer, J.M.; Miranda, P.; Vencovský, J.; Godwood, A.; Albulescu, M.; Michaels, M.A.; Guo, X.; Close, D.; et al. Mavrilimumab, a Fully Human Granulocyte-Macrophage Colony-Stimulating Factor Receptor α Monoclonal Antibody: Long-Term Safety and Efficacy in Patients With Rheumatoid Arthritis. *Arthritis Rheumatol.***2018**, *70*, 679–689, doi:10.1002/art.40420.

89. Kiniksa Pharmaceuticals Ltd. Kiniksa Announces Early Evidence of Treatment Response with Mavrilimumab in 6 Patients with Severe COVID-19 Pneumonia and Hyperinflammation. https://www.globenewswire.com/news-release/2020/03/31/2009126/0/en/Kiniksa-Announces-Early-Evidence-of-Treatment-Response-with-Mavrilimumab-in-6-Patients-with-Severe-COVID-19-Pneumonia-and-Hyperinflammation.html (accessed on 15 April 2020).

90. Wang, Y.; Jiang, W.; He, Q.; Wang, C.; Wang, B.; Zhou, P.; Dong, N.; Tong, Q. Early, low-dose and short-term application of corticosteroid treatment in patients with severe COVID-19 pneumonia: single-center experience from Wuhan, China. medRxiv doi: 10.1101/2020.03.06.20032342. https://www.medrxiv.org/content/10.1101/2020.03.06.20032342v1 (accessed on 15 April 2020).

91. Zheng, W.; Fan, W.; Zhang, S.; Jiao, P.; Shang, Y.; Cui, L.; Mahesutihan, M.; Li, J.; Wang, D.; Gao, G.F.; et al. Naproxen Exhibits Broad Anti-influenza Virus Activity in Mice by Impeding Viral Nucleoprotein Nuclear Export. *Cell Rep.***2019**, *27*, 1875-1885.e5, doi:10.1016/j.celrep.2019.04.053.

92. Flaherty, K.R.; Wells, A.U.; Cottin, V.; Devaraj, A.; Walsh, S.L.F.; Inoue, Y.; Richeldi, L.; Kolb, M.; Tetzlaff, K.; Stowasser, S.; et al. Nintedanib in Progressive Fibrosing Interstitial Lung Diseases. *N. Engl. J. Med.***2019**, *381*, 1718–1727, doi:10.1056/NEJMoa1908681.

93. Richeldi, L.; Du Bois, R.M.; Raghu, G.; Azuma, A.; Brown, K.K.; Costabel, U.; Cottin, V.; Flaherty, K.R.; Hansell, D.M.; Inoue, Y.; et al. Efficacy and safety of nintedanib in idiopathic pulmonary fibrosis. *N. Engl. J. Med.***2014**, *370*, 2071–2082, doi:10.1056/NEJMoa1402584.

94. Rossignol, J.-F. Nitazoxanide: a first-in-class broad-spectrum antiviral agent. *Antiviral Res.***2014**, *110*, 94–103, doi:10.1016/j.antiviral.2014.07.014.

95. Hong, S.K.; Kim, H.J.; Song, C.S.; Choi, I.S.; Lee, J.B.; Park, S.Y. Nitazoxanide suppresses IL-6 production in LPS-stimulated mouse macrophages and TG-injected mice. *Int. Immunopharmacol.***2012**, *13*, 23–27, doi:10.1016/j.intimp.2012.03.002.

96. Kelleni, M.T. Nitazoxanide/azithromycin combination for COVID-19: A suggested new protocol for early management. *Pharmacol. Res.***2020**, *157*, 104874, doi:10.1016/j.phrs.2020.104874.

97. Yu, B.; Ichinose, F.; Bloch, D.B.; Zapol, W.M. Inhaled nitric oxide. *Br. J. Pharmacol.***2019**, *176*, 246–255, doi:10.1111/bph.14512.

98. Akerström, S.; Gunalan, V.; Keng, C.T.; Tan, Y.-J.; Mirazimi, A. Dual effect of nitric oxide on SARS-CoV replication: viral RNA production and palmitoylation of the S protein are affected. *Virology***2009**, *395*, 1–9, doi:10.1016/j.virol.2009.09.007.

99. Keyaerts, E.; Vijgen, L.; Chen, L.; Maes, P.; Hedenstierna, G.; van Ranst, M. Inhibition of SARS-coronavirus infection in vitro by S-nitroso-N-acetylpenicillamine, a nitric oxide donor compound. *Int. J. Infect. Dis.***2004**, *8*, 223–226, doi:10.1016/j.ijid.2004.04.012.

100. Simsek, M.; Tekin, S.B.; Bilici, M. Immunological Agents Used in Cancer Treatment. *Eurasian J. Med.***2019**, *51*, 90–94, doi:10.5152/eurasianjmed.2018.18194.

101. Welliver, R.; Monto, A.S.; Carewicz, O.; Schatteman, E.; Hassman, M.; Hedrick, J.; Jackson, H.C.; Huson, L.; Ward, P.; Oxford, J.S. Effectiveness of oseltamivir in preventing influenza in household contacts: a randomized controlled trial. *JAMA***2001**, *285*, 748–754, doi:10.1001/jama.285.6.748.

102. Mitjà, O.; Clotet, B. Use of antiviral drugs to reduce COVID-19 transmission. *Lancet Glob. Health***2020**, doi:10.1016/S2214-109X(20)30114-5.

103. Hemann, E.A.; Gale, M.; Savan, R. Interferon Lambda Genetics and Biology in Regulation of Viral Control. *Front. Immunol.***2017**, *8*, 1707, doi:10.3389/fimmu.2017.01707.

104. Mordstein, M.; Neugebauer, E.; Ditt, V.; Jessen, B.; Rieger, T.; Falcone, V.; Sorgeloos, F.; Ehl, S.; Mayer, D.; Kochs, G.; et al. Lambda interferon renders epithelial cells of the respiratory and gastrointestinal tracts resistant to viral infections. *J. Virol.***2010**, *84*, 5670–5677, doi:10.1128/JVI.00272-10.

105. Lee, H.T.; Lee, S.H.; Heo, Y.-S. Molecular Interactions of Antibody Drugs Targeting PD-1, PD-L1, and CTLA-4 in Immuno-Oncology. *Molecules***2019**, *24*, doi:10.3390/molecules24061190.

106. Fessas, P.; Lee, H.; Ikemizu, S.; Janowitz, T. A molecular and preclinical comparison of the PD-1-targeted T-cell checkpoint inhibitors nivolumab and pembrolizumab. *Semin. Oncol.***2017**, *44*, 136–140, doi:10.1053/j.seminoncol.2017.06.002.

107. Middleton, G.; Brock, K.; Savage, J.; Mant, R.; Summers, Y.; Connibear, J.; Shah, R.; Ottensmeier, C.; Shaw, P.; Lee, S.-M.; et al. Pembrolizumab in patients with non-small-cell lung cancer of performance status 2 (PePS2): a single arm, phase 2 trial. *Lancet Respir. Med.***2020**, doi:10.1016/S2213-2600(20)30033-3.

108. Fishman, P.; Cohen, S. The A3 adenosine receptor (A3AR): therapeutic target and predictive biological marker in rheumatoid arthritis. *Clin. Rheumatol.***2016**, *35*, 2359–2362, doi:10.1007/s10067-016-3202-4.

109. Woo, Y.R.; Cho, D.H.; Park, H.J. Molecular Mechanisms and Management of a Cutaneous Inflammatory Disorder: Psoriasis. *Int. J. Mol. Sci.***2017**, *18*, doi:10.3390/ijms18122684.

110. Gin, A.; Dilay, L.; Karlowsky, J.A.; Walkty, A.; Rubinstein, E.; Zhanel, G.G. Piperacillin-tazobactam: a beta-lactam/beta-lactamase inhibitor combination. *Expert Rev. Anti Infect. Ther.***2007**, *5*, 365–383, doi:10.1586/14787210.5.3.365.

111. Peng, X.; Xu, X.; Li, Y.; Cheng, L.; Zhou, X.; Ren, B. Transmission routes of 2019-nCoV and controls in dental practice. *Int. J. Oral Sci.***2020**, *12*, 9, doi:10.1038/s41368-020-0075-9.

112. Shafiq, H.B.; Amin, U.; Nawaz, S. Comparative analysis of various antimicrobial agents present in locally available mouthwashes against oral pathogens. *Pak. J. Pharm. Sci.***2018**, *31*, 1881–1887.

113. Flynn, J. Povidone-iodine as a topical antiseptic for treating and preventing wound infection: a literature review. *Br. J. Community. Nurs.***2003**, *8*, S36-42, doi:10.12968/bjcn.2003.8.Sup2.11555.

114. Durani, P.; Leaper, D. Povidone-iodine: use in hand disinfection, skin preparation and antiseptic irrigation. *Int. Wound J.***2008**, *5*, 376–387, doi:10.1111/j.1742-481X.2007.00405.x.

115. Liu, D.; Ahmet, A.; Ward, L.; Krishnamoorthy, P.; Mandelcorn, E.D.; Leigh, R.; Brown, J.P.; Cohen, A.; Kim, H. A practical guide to the monitoring and management of the complications of systemic corticosteroid therapy. *Allergy Asthma Clin. Immunol.***2013**, *9*, 30, doi:10.1186/1710-1492-9-30.

116. Gordon, C.J.; Tchesnokov, E.P.; Woolner, E.; Perry, J.K.; Feng, J.Y.; Porter, D.P.; Gotte, M. Remdesivir is a direct-acting antiviral that inhibits RNA-dependent RNA polymerase from severe acute respiratory syndrome coronavirus 2 with high potency. *J. Biol. Chem.***2020**, doi:10.1074/jbc.RA120.013679.

117. Amirian, E.S.; Levy, J.K. Current knowledge about the antivirals remdesivir (GS-5734) and GS-441524 as therapeutic options for coronaviruses. *One Health***2020**, *9*, 100128, doi:10.1016/j.onehlt.2020.100128.

118. Falzarano, D.; Wit, E. de; Rasmussen, A.L.; Feldmann, F.; Okumura, A.; Scott, D.P.; Brining, D.; Bushmaker, T.; Martellaro, C.; Baseler, L.; et al. Treatment with interferon-α2b and ribavirin improves outcome in MERS-CoV-infected rhesus macaques. *Nat. Med.***2013**, *19*, 1313–1317, doi:10.1038/nm.3362.

119. Kim, U.J.; Won, E.-J.; Kee, S.-J.; Jung, S.-I.; Jang, H.-C. Combination therapy with lopinavir/ritonavir, ribavirin and interferon-α for Middle East respiratory syndrome. *Antivir. Ther. (Lond.)***2016**, *21*, 455–459, doi:10.3851/IMP3002.

120. Pawlotsky, J.-M.; Dahari, H.; Neumann, A.U.; Hezode, C.; Germanidis, G.; Lonjon, I.; Castera, L.; Dhumeaux, D. Antiviral action of ribavirin in chronic hepatitis C. *Gastroenterology* **2004**, *126*, 703–714, doi:10.1053/j.gastro.2003.12.002.

121. Mondelli, M.U. The multifaceted functions of ribavirin: antiviral, immunomodulator, or both? *Hepatology* **2014**, *60*, 1126–1129, doi:10.1002/hep.27186.

122. Lv, Z.; Chu, Y.; Wang, Y. HIV protease inhibitors: a review of molecular selectivity and toxicity. *HIV AIDS (Auckl)* **2015**, *7*, 95–104, doi:10.2147/HIV.S79956.

123. Stellbrink, H.-J.; Arastéh, K.; Schürmann, D.; Stephan, C.; Dierynck, I.; Smyej, I.; Hoetelmans, R.M.W.; Truyers, C.; Meyvisch, P.; Jacquemyn, B.; et al. Antiviral activity, pharmacokinetics, and safety of the HIV-1 protease inhibitor TMC310911, coadministered with ritonavir, in treatment-naive HIV-1-infected patients. *J. Acquir. Immune Defic. Syndr.* **2014**, *65*, 283–289, doi:10.1097/QAI.0000000000000003.

124. Hoetelmans, R.M.W.; Dierynck, I.; Smyej, I.; Meyvisch, P.; Jacquemyn, B.; Marien, K.; Simmen, K.; Verloes, R. Safety and pharmacokinetics of the HIV-1 protease inhibitor TMC310911 coadministered with ritonavir in healthy participants: results from 2 phase 1 studies. *J. Acquir. Immune Defic. Syndr.* **2014**, *65*, 299–305, doi:10.1097/QAI.0000000000000011.

125. Markham, A.; Keam, S.J. Danoprevir: First Global Approval. *Drugs* **2018**, *78*, 1271–1276, doi:10.1007/s40265-018-0960-0.

126. Zu, Z.Y.; Di Jiang, M.; Xu, P.P.; Chen, W.; Ni, Q.Q.; Lu, G.M.; Zhang, L.J. Coronavirus Disease 2019 (COVID-19): A Perspective from China. *Radiology* **2020**, 200490, doi:10.1148/radiol.2020200490.

127. Lu, R.; Zhao, X.; Li, J.; Niu, P.; Yang, B.; Wu, H.; Wang, W.; Song, H.; Huang, B.; Zhu, N.; et al. Genomic characterisation and epidemiology of 2019 novel coronavirus: implications for virus origins and receptor binding. *Lancet* **2020**, *395*, 565–574, doi:10.1016/S0140-6736(20)30251-8.

128. Chen, Y.; Liu, Q.; Guo, D. Emerging coronaviruses: Genome structure, replication, and pathogenesis. *J. Med. Virol.* **2020**, *92*, 418–423, doi:10.1002/jmv.25681.

129. Spiegel, M.; Pichlmair, A.; Mühlberger, E.; Haller, O.; Weber, F. The antiviral effect of interferon-beta against SARS-coronavirus is not mediated by MxA protein. *J. Clin. Virol.* **2004**, *30*, 211–213, doi:10.1016/j.jcv.2003.11.013.

130. Zeng, Y.-M.; Xu, X.-L.; He, X.-Q.; Tang, S.-Q.; Li, Y.; Huang, Y.-Q.; Harypursat, V.; Chen, Y.-K. Comparative effectiveness and safety of ribavirin plus interferon-alpha, lopinavir/ritonavir plus interferon-alpha and ribavirin plus lopinavir/ritonavir plus interferon-alphain in patients with mild to moderate novel coronavirus pneumonia. *Chin. Med. J.* **2020**, doi:10.1097/CM9.0000000000000790.

131. Hart, R.G.; Sharma, M.; Mundl, H.; Kasner, S.E.; Bangdiwala, S.I.; Berkowitz, S.D.; Swaminathan, B.; Lavados, P.; Wang, Y.; Wang, Y.; et al. Rivaroxaban for Stroke Prevention after Embolic Stroke of Undetermined Source. *N. Engl. J. Med.* **2018**, *378*, 2191–2201, doi:10.1056/NEJMoa1802686.

132. Bratsos, S. Pharmacokinetic Properties of Rivaroxaban in Healthy Human Subjects. *Cureus* **2019**, *11*, e5484, doi:10.7759/cureus.5484.

133. Waller, E.K. The role of sargramostim (rhGM-CSF) as immunotherapy. *Oncologist* **2007**, *12 Suppl 2*, 22–26, doi:10.1634/theoncologist.12-S2-22.

134. Sanofi; Regeneron Pharmaceuticals Inc. Sanofi and Regeneron begin global Kevzara® (sarilumab) clinical trial program in patients with severe COVID-19. http://www.news.sanofi.us/2020-03-16-Sanofi-and-Regeneron-begin-global-Kevzara-R-sarilumab-clinical-trial-program-in-patients-with-severe-COVID-19 (accessed on 15 April 2020).

135. Barnett, C.F.; Machado, R.F. Sildenafil in the treatment of pulmonary hypertension. *Vasc. Health Risk. Manag.* **2006**, *2*, 411–422, doi:10.2147/vhrm.2006.2.4.411.

136. Karkera, J.; Steiner, H.; Li, W.; Skradski, V.; Moser, P.L.; Riethdorf, S.; Reddy, M.; Puchalski, T.; Safer, K.; Prabhakar, U.; et al. The anti-interleukin-6 antibody siltuximab down-regulates genes implicated in tumorigenesis in prostate cancer patients from a phase I study. *Prostate* **2011**, *71*, 1455–1465, doi:10.1002/pros.21362.

137. Fedson, D.S. Pandemic influenza: a potential role for statins in treatment and prophylaxis. *Clin. Infect. Dis.* **2006**, *43*, 199–205, doi:10.1086/505116.

138. Bowman, L.J.; Brueckner, A.J.; Doligalski, C.T. The Role of mTOR Inhibitors in the Management of Viral Infections: A Review of Current Literature. *Transplantation* **2018**, *102*, S50-S59, doi:10.1097/TP.0000000000001777.

139. Liao, W.; Ma, X.; Li, J.; Li, X.; Guo, Z.; Zhou, S.; Sun, H. A review of the mechanism of action of Dantonic® for the treatment of chronic stable angina. *Biomed. Pharmacother.* **2019**, *109*, 690–700, doi:10.1016/j.biopha.2018.10.013.

140. Iwasaki, K. Metabolism of tacrolimus (FK506) and recent topics in clinical pharmacokinetics. *Drug Metab. Pharmacokinet.* **2007**, *22*, 328–335, doi:10.2133/dmpk.22.328.

141. Yamagishi, S.-I.; Nakamura, K.; Matsui, T. Potential utility of telmisartan, an angiotensin II type 1 receptor blocker with peroxisome proliferator-activated receptor-gamma (PPAR-gamma)-modulating activity for the treatment of cardiometabolic disorders. *Curr. Mol. Med.* **2007**, *7*, 463–469, doi:10.2174/156652407781387073.

142. Rothlin, R.P.; Vetulli, H.M.; Duarte, M.; Pelorosso, F.G. Telmisartan as tentative angiotensin receptor blocker therapeutic for COVID-19. *Drug Dev. Res.* **2020**, doi:10.1002/ddr.21679.

143. Battershill, A.J.; Scott, L.J. Telmisartan: a review of its use in the management of hypertension. *Drugs* **2006**, *66*, 51–83, doi:10.2165/00003495-200666010-00004.

144. Zhu, H.; Shi, X.; Ju, D.; Huang, H.; Wei, W.; Dong, X. Anti-inflammatory effect of thalidomide on H1N1 influenza virus-induced pulmonary injury in mice. *Inflammation* **2014**, *37*, 2091–2098, doi:10.1007/s10753-014-9943-9.

145. Zhao, L.; Xiao, K.; Wang, H.; Wang, Z.; Sun, L.; Zhang, F.; Zhang, X.; Tang, F.; He, W. Thalidomide has a therapeutic effect on interstitial lung fibrosis: evidence from in vitro and in vivo studies. *Clin. Exp. Immunol.* **2009**, *157*, 310–315, doi:10.1111/j.1365-2249.2009.03962.x.

146. Kwon, H.Y.; Han, Y.J.; Im, J.H.; Baek, J.H.; Lee, J.-S. Two cases of immune reconstitution inflammatory syndrome in HIV patients treated with thalidomide. *Int. J. STD AIDS* **2019**, *30*, 1131–1135, doi:10.1177/0956462419847297.

147. Le, R.Q.; Li, L.; Yuan, W.; Shord, S.S.; Nie, L.; Habtemariam, B.A.; Przepiorka, D.; Farrell, A.T.; Pazdur, R. FDA Approval Summary: Tocilizumab for Treatment of Chimeric Antigen Receptor T Cell-Induced Severe or Life-Threatening Cytokine Release Syndrome. *Oncologist* **2018**, *23*, 943–947, doi:10.1634/theoncologist.2018-0028.

148. Morrondo, C.D.; Zarza, L.P.; Gil, J.G.; Pinto Tasende, J.A.; Diez, P.D.; López, J.M.B. Benefit of Tocilizumab Therapy for Adult-Onset Still Disease Complicated With Acute Respiratory Distress Syndrome. *J. Clin. Rheumatol.* **2016**, *22*, 291–293, doi:10.1097/RHU.0000000000000374.

149. Choy, E.H. Clinical significance of Janus Kinase inhibitor selectivity. *Rheumatology (Oxford)* **2019**, *58*, 953–962, doi:10.1093/rheumatology/key339.

150. Schwartz, D.M.; Kanno, Y.; Villarino, A.; Ward, M.; Gadina, M.; O'Shea, J.J. JAK inhibition as a therapeutic strategy for immune and inflammatory diseases. *Nat. Rev. Drug Discov.* **2017**, *17*, 78, doi:10.1038/nrd.2017.267.

151. McInnes, I.B.; Byers, N.L.; Higgs, R.E.; Lee, J.; Macias, W.L.; Na, S.; Ortmann, R.A.; Rocha, G.; Rooney, T.P.; Wehrman, T.; et al. Comparison of baricitinib, upadacitinib, and tofacitinib mediated regulation of cytokine signaling in human leukocyte subpopulations. *Arthritis Res. Ther.* **2019**, *21*, 183, doi:10.1186/s13075-019-1964-1.

152. Vyas, D.; O'Dell, K.M.; Bandy, J.L.; Boyce, E.G. Tofacitinib: The First Janus Kinase (JAK) inhibitor for the treatment of rheumatoid arthritis. *Ann. Pharmacother.* **2013**, *47*, 1524–1531, doi:10.1177/1060028013512790.

153. Drug.Bank.ca. Tradipitant. https://www.drugbank.ca/drugs/DB12580 (accessed on 15 April 2020).

154. Diop, A.; Abbas, D.; Ngoc, N.t.N.; Martin, R.; Razafi, A.; Tuyet, H.T.D.; Winikoff, B. A double-blind, randomized controlled trial to explore oral tranexamic acid as adjunct for the treatment for postpartum hemorrhage. *Reprod. Health* **2020**, *17*, doi:10.1186/s12978-020-0887-2.

155. Blaising, J.; Polyak, S.J.; Pécheur, E.-I. Arbidol as a broad-spectrum antiviral: An update. *Antiviral Res.* **2014**, *107*, 84–94, doi:10.1016/j.antiviral.2014.04.006.

156. Haviernik, J.; Štefánik, M.; Fojtíková, M.; Kali, S.; Tordo, N.; Rudolf, I.; Hubálek, Z.; Eyer, L.; Ruzek, D. Arbidol (Umifenovir): A Broad-Spectrum Antiviral Drug That Inhibits Medically Important Arthropod-Borne Flaviviruses. *Viruses* **2018**, *10*, 184, doi:10.3390/v10040184.

157. Harrison, C. Coronavirus puts drug repurposing on the fast track. *Nat. Biotechnol.* **2020**, *38*, 379–381, doi:10.1038/d41587-020-00003-1.

158. Dandona, P.; Dhindsa, S.; Ghanim, H.; Chaudhuri, A. Angiotensin II and inflammation: the effect of angiotensin-converting enzyme inhibition and angiotensin II receptor blockade. *J. Hum. Hypertens.* **2007**, *21*, 20–27, doi:10.1038/sj.jhh.1002101.

159. Black, H.R.; Bailey, J.; Zappe, D.; Samuel, R. Valsartan: more than a decade of experience. *Drugs***2009**, *69*, 2393–2414, doi:10.2165/11319460-000000000-00000.
